# Intracellular Events and Cell Fate in Filovirus Infection

**DOI:** 10.3390/v3081501

**Published:** 2011-08-24

**Authors:** Judith Olejnik, Elena Ryabchikova, Ronald B. Corley, Elke Mühlberger

**Affiliations:** 1 Department of Microbiology, School of Medicine, Boston University, 72 East Concord Street, Boston, MA 02118, USA; E-Mails: jolejnik@bu.edu (J.O.); rbcorley@bu.edu (R.B.C.); 2 National Emerging Infectious Diseases Laboratories Institute, Boston University, 72 East Concord Street, Boston, MA 02118, USA; 3 Institute of Chemical Biology and Fundamental Medicine, Siberian Branch of Russian Academy of Science, Pr. Lavrent’eva, 8, Novosibirsk 630090, Russian Federation; E-Mail: lenryab@yandex.ru

**Keywords:** Ebola Virus, Marburg Virus, filoviruses, viral replication cycle, target cells, animal models, ultrastructural analysis, virus-cell interaction, bystander apoptosis, cell death

## Abstract

Marburg and Ebola viruses cause a severe hemorrhagic disease in humans with high fatality rates. Early target cells of filoviruses are monocytes, macrophages, and dendritic cells. The infection spreads to the liver, spleen and later other organs by blood and lymph flow. A hallmark of filovirus infection is the depletion of non-infected lymphocytes; however, the molecular mechanisms leading to the observed bystander lymphocyte apoptosis are poorly understood. Also, there is limited knowledge about the fate of infected cells in filovirus disease. In this review we will explore what is known about the intracellular events leading to virus amplification and cell damage in filovirus infection. Furthermore, we will discuss how cellular dysfunction and cell death may correlate with disease pathogenesis.

## 1. Introduction

The members of the filovirus family, Ebola virus (EBOV) and Marburg virus (MARV), cause a severe hemorrhagic fever in infected humans with high fatality rates [1]. Infected individuals who go on to succumb to filovirus infection exhibit dysregulated immune responses (reviewed in two other articles in this issue). This appears to result from several factors, including viral mediated impairment of early innate immune responses and consequent dysregulation of innate immunity [2]. Some reports have suggested that adaptive immune responses may occur [3–6], but it is evident that these fail to clear the disease. Lymphopenia resulting from apoptosis as the infection progresses has also been suggested to contribute to the failure to clear the infection [7]. Studies of both human survivors and murine model systems suggest that a well-regulated cytokine response early in the course of the infection may be critical to the outcome of the disease [8,9].

Ebolaviruses are currently subdivided into four distinct species, *Zaire ebolavirus* (ZEBOV), *Sudan ebolavirus*, *Tai Forest ebolavirus*, and *Reston ebolavirus* (REBOV), while there is only a single MARV species (*Lake Victoria marburgvirus)* (ICTV virus taxonomy 2009). Since the Bundibugyo isolate is genetically distinct from the known Ebola viruses, a suggestion has been made to classify it as a new EBOV species, *Bundibugyo ebolavirus* [10]. The different EBOV species not only show significant molecular differences, they also vary in terms of virulence and pathogenicity. The most pathogenic species in humans is ZEBOV with a case fatality rate of about 80%, followed by Sudan with a case fatality rate of about 50% [11], and Bundibugyo with a fatality rate of about 30% [12]. To date, there are two reported non-fatal human cases of Tai Forest ebolavirus [13,14] and several asymptomatic human cases of REBOV infection [15–17].

The first reported MARV outbreak occurred in Germany and Yugoslavia in 1967 and was caused by infected African green monkeys imported from Uganda [18,19]. Since this outbreak was associated with a case fatality rate of 22%, it was believed for a long time that MARV was less pathogenic than EBOV. However, recent outbreaks of MARV in the Democratic Republic of the Congo in 1998–2000 and in Angola in 2004 were associated with fatality rates up to 90%, indicating that MARV can be as virulent as EBOV [20–22].

Despite the severity of the disease, filoviruses have been regarded as exotic pathogens with fatal outbreaks restricted to Central Africa, and with no major health threat outside of the endemic areas. Knowledge on their biology and pathogenicity consequently remained limited. However, there has been renewed interest given the potential for using filoviruses in bioterrorism attacks and the possibility for infected, asymptomatic persons for bringing the disease to other countries. Indeed, two cases of MARV have been reported in the Netherlands and in the United States, both tourists returning from trips to Uganda [23,24]. Together, the potential for spread outside central Africa has reignited research endeavors to elucidate the biology of the filoviruses and to develop effective therapeutic strategies.

In this review we will describe how filoviruses enter their target cells, replicate their genomes and assemble progeny viruses by exploiting cellular machineries. We will also briefly touch upon the interaction of filoviruses with cellular signaling pathways. Finally, we will discuss the current understanding of the fate of infected and non-infected cells in filovirus infection. In addition, we will present ultrastructural data of infected and non-infected cells, demonstrating the morphological changes in filovirus infection.

## 2. The Central Players: Virus and Target Cells

### 2.1. Filovirus Structure

The structure of filovirus particles has been described in detail in [1] and multiple review articles. Briefly, the single stranded non-segmented RNA genome of filoviruses is of negative polarity and contains seven monocistronic genes. It is associated with four viral proteins, the nucleoprotein NP which enwraps the viral RNA, the RNA-dependent RNA polymerase L, the polymerase cofactor VP35, and the transcription factor VP30. The four nucleocapsid proteins are required for replication and transcription of the viral genome (reviewed in [25]). Filovirus genomes encode two matrix proteins, VP40, the functional equivalent of the matrix proteins M of other non-segmented negative-stand RNA viruses, and the minor matrix protein, VP24, which is unique to filoviruses. As a peripheral membrane protein, VP40 is located at the inner side of the virion’s membrane. It mediates budding and viral particle release [26]. The minor matrix protein VP24 is involved in nucleocapsid formation and assembly [27–29] and contributes to the regulation of viral transcription/replication [30,31]. EBOV VP24 and MARV VP40 are considered important virulence factors and play a crucial role in host adaptation. Both proteins block IFN signaling, however, they target different cellular proteins and use different mechanisms to antagonize the IFN response [32–35]. The role of EBOV VP24 and MARV VP40 in the innate immune response to filovirus infection will be discussed in more detail in another article in this issue. Filoviruses possess a single surface protein, the type I transmembrane glycoprotein GP that mediates attachment to target cells, entry, and fusion [36]. The precursor preGP is cleaved in the trans Golgi network by furin or a furin-like protease resulting in two disulfide-linked subunits, GP_1_and GP_2_ [37]. Notably, EBOV GP has been implicated in cell damage, which will be discussed in more detail in Section 4.2.2.

EBOV genomes encode an additional protein, the nonstructural soluble form of the glycoprotein, sGP. As GP, sGP is encoded by the fourth gene, but is translated from non-edited mRNA species, while the membrane-bound GP is the result of mRNA editing during transcription [38,39]. sGP is not incorporated into viral particles, but is secreted from infected cells. Although the function of the protein is not fully understood, there is evidence that it acts as an anti-inflammatory factor by protecting the endothelial cell barrier function during infection [40]. Besides sGP, a second soluble GP variant generated by mRNA editing, the small soluble protein (ssGP) has been identified [41,42]. A nonstructural MARV protein comparable to EBOV sGP is not expressed.

### 2.2. Target Cells in Filovirus Infection

Filoviruses have a broad cell tropism in susceptible host species. Among the target cells supporting viral replication are monocytes, macrophages, dendritic cells (DCs), hepatocytes, adrenal cortical cells, fibroblasts and endothelial cells [43–57].

The earliest events during infection are likely to center around cells of the mononuclear phagocyte system, including monocytes, macrophages, and DCs. These cells not only orchestrate innate and adaptive immune responses [58,59], but also serve as early targets of viral infection [47–49,55,56, 60–66]. It is thought that the early infection of these cells is responsible for the rapid and widespread dissemination of the virus throughout an infected host [67].

Replication of MARV in peritoneal macrophages of guinea pigs can be detected within 24 h after intraperitoneal infection [50]. In African green monkeys, infected Kupffer cells were identified in the liver early in infection, on days 2 or 3 following infection with ZEBOV or MARV. The liver and adrenals are target organs of these viruses, whose parenchyma cells support filovirus replication. Infected hepatocytes and adrenal cortical cells can be detected a day or two after finding infected macrophages. Secondary target cells also include fibroblasts and endothelial cells, which were observed 5 to 7 days after filovirus infection of non-human primates [47,49,50]. Filovirus-infected macrophages, fibroblasts, and endothelial cells were found in all organs examined in these animal models [46–52,55]. The presence of these cell types in virtually all organs may account for the observed filovirus pantropism. Indeed, MARV or ZEBOV can be isolated from any organ or tissue. Besides the typical target cells for ZEBOV and MARV infection in non-human primates, additional target cells were occasionally found in individual animals. These cells included alveolar epithelial cells, bronchial epithelial cells and the cells of endocardial layer [50]. In cynomolgus macaques, REBOV was shown to have similar target tropism, and also to rarely infect alveolar and kidney epithelium as well as adrenal medulla cells [64].

Filoviruses are present in the blood of infected animals and are therefore potentially spread to all parts of the body by blood flow both as free virions as well as within infected monocytes. It is likely that the lymphatics also contribute to the rapid spread of the virus as free viruses and virus-infected DCs. The structure of sinusoids and sinuses in the liver and spleen allows for the direct migration of filoviruses from the blood stream, facilitating the infection of hepatocytes and splenic macrophages. Migration of infected monocytes from blood vessels may deliver the virus into connective tissue where it infects fibroblasts, which then spread progeny viruses by their protrusions to sites far from the main body of the infected cell [46,49,50,68]. It is therefore likely that filoviruses are disseminated in the infected host by multiple different mechanisms including transport of free virus particles by blood and lymphatic fluids, migration of infected monocytes, macrophages and DCs into various tissues, and viral cell-to-cell spread via cell protrusions.

One of the most intriguing features of fatal filovirus infection in animals and humans is that little or no inflammatory cellular response occurs at the sites of viral replication. Accumulation of neutrophils, monocytes, and lymphocytes around infected cells has been rarely observed in infected tissues. The minimal inflammatory cellular response is considered to be a distinctive feature of filovirus infection [45,49,55,60,63,69–71] and may represent a part of the dysregulated immune response observed in fatal cases of EBOV and MARV infection. In non-fatal EBOV infections of guinea pigs, in contrast, a prominent inflammatory response is observed, and infected cells are tightly surrounded by leukocytes forming a substantial barrier which could impair viral dissemination [48,62].

## 3. What Happens in the Infected Cell?

### 3.1. Entry: Speak Friend and Enter

The first step that determines if a cell will be a target for infection is the ability of the virus to attach. Filoviruses are able to infect multiple cell types *in vivo* and in cell culture by exploiting cellular entry machineries. However, some cells are more susceptible to infection than others, and some are not permissive to infection.

**Figure 1.**
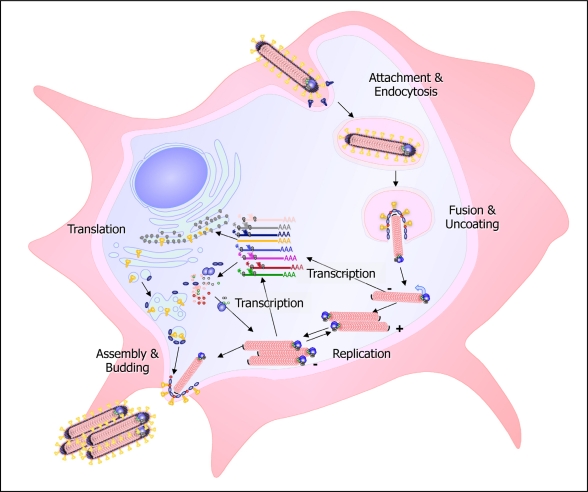
Scheme of the filovirus infection cycle. Filoviruses enter the cell by receptor-mediated endocytosis or macropinocytosis. After fusion of the viral and cellular membrane, the nucleocapsid is released into the cytoplasm and serves as a template for transcription and replication. The replicated RNA is encapsidated by the nucleocapsid proteins. The newly synthesized nucleocapsids are transported to the sites of viral release, where budding takes place.

The filovirus replication cycle is depicted in Figure 1. Filoviruses enter the target cells by different uptake mechanisms including lipid raft-dependent and receptor-mediated endocytosis [72–75] and macropinocytosis [76,77]. Receptor binding and attachment to the target cells is mediated by the glycoprotein subunit GP_1_. A number of cellular proteins have been implicated in filovirus entry, and it is not clear whether there is a “primary” receptor for these viruses. The different co-receptors likely provide access for the virus into different target cells. The folate receptor has been suggested to be a significant filovirus receptor [78]. However, cells lacking folate receptor-α are still permissive for infection [79,80]. An interesting group of glycan binding proteins that enhance filovirus uptake in a cell-specific manner belong to the C-type lectin family. The highly glycosylated GP of both MARV and EBOV is decorated with a set of N- and O-linked glycans [81–83] which, depending on their specific structures, can be recognized by different C-type lectins. This includes asialoglycoprotein receptor on hepatocytes, DC-SIGN and hMGL on macrophages and immature DCs, and L-SIGN and LSECtin on endothelial cells in liver and lymph nodes [84–91]. Another group of proteins involved in filovirus entry are the β_1_-integrins [92,93]. Integrins are expressed on a wide range of cell types and are involved in the uptake of a variety of different viruses. Interestingly, detailed study of one of these integrins, the α_5_β_1_-integrin, has demonstrated that it is not involved in EBOV internalization, but rather in the regulation of endosomal cathepsin required for EBOV fusion [93]. More recently T-cell immunoglobulin and mucin domain 1 (TIM-1) has been suggested as a receptor for EBOV and MARV GP [94]. TIM-1 is not expressed by the primary targets of filoviruses, macrophages and DCs, but is expressed on mucosal epithelial cells, whose role in infection is not clear yet. Another interesting group of proteins involved in EBOV uptake are the members of the Tyro3/Axl/Mer (TAM) receptor family. Ligand-activated TAM receptors are negative regulators of inflammation in macrophages and DCs by upregulating the expression of SOCS1 and SOCS3 proteins in a phospho-STAT1-dependent manner [95]. The TAM receptor Axl serves as a co-receptor for EBOV entry by binding the viral surface protein GP [96]. To bind GP, the TAM receptor ligand binding domain has to be intact [97]. However, it is not known if GP binding leads to the activation of Axl. Recently, Axl has been shown to be involved in receptor-independent uptake of EBOV by macropinocytosis [75].

After uptake, the virus particles are internalized into the endosomes, where fusion takes place. Fusion of the viral and cellular membrane is mediated by the fusogenic cleavage product GP_2_ [98]. To initiate fusion, the proteolytic cleavage of GP_1_ by the endosomal proteases cathepsin B and cathepsin L is mandatory [99–101]. Interestingly, the cathepsin dependence of virus entry seems to be cell-type specific. While virus entry into Vero cells is dependent on the activity of both cathepsin B and cathepsin L, infection of human DCs by EBOV does not require active cathepsin L [102].

### 3.2. Replication: The Intruders Take Over

Fusion of the viral and cellular membrane leads to the release of the viral nucleocapsids into the cytoplasm of the infected cell where transcription and replication of the viral genome take place (Figure 1). The nucleocapsid, rather than the naked RNA, serves as the template for both transcription and replication. During transcription, the seven viral genes are sequentially transcribed into monocistronic mRNAs which are capped and polyadenylated and are used for the production of viral proteins. During replication, the encapsidated RNA is copied into full-length positive-sense replicative intermediates, the RNA antigenomes, which are enwrapped by the nucleocapsid proteins. In turn, the antigenomes are used as templates for the synthesis of progeny genomes. The nucleocapsid proteins do not only encapsidate the RNA genomes, they are also essential for replication and transcription. The viral polymerase, consisting of L and VP35, catalyzes replication as well as transcription, including polyadenylation and capping (reviewed in [25]).

The driving force for nucleocapsid formation is NP [103,104]. It has been proposed for EBOV that NP forms helical structures which interact with VP35 and VP24, resulting in the formation of nucleocapsid-like structures [105,106].

**Figure 2.**
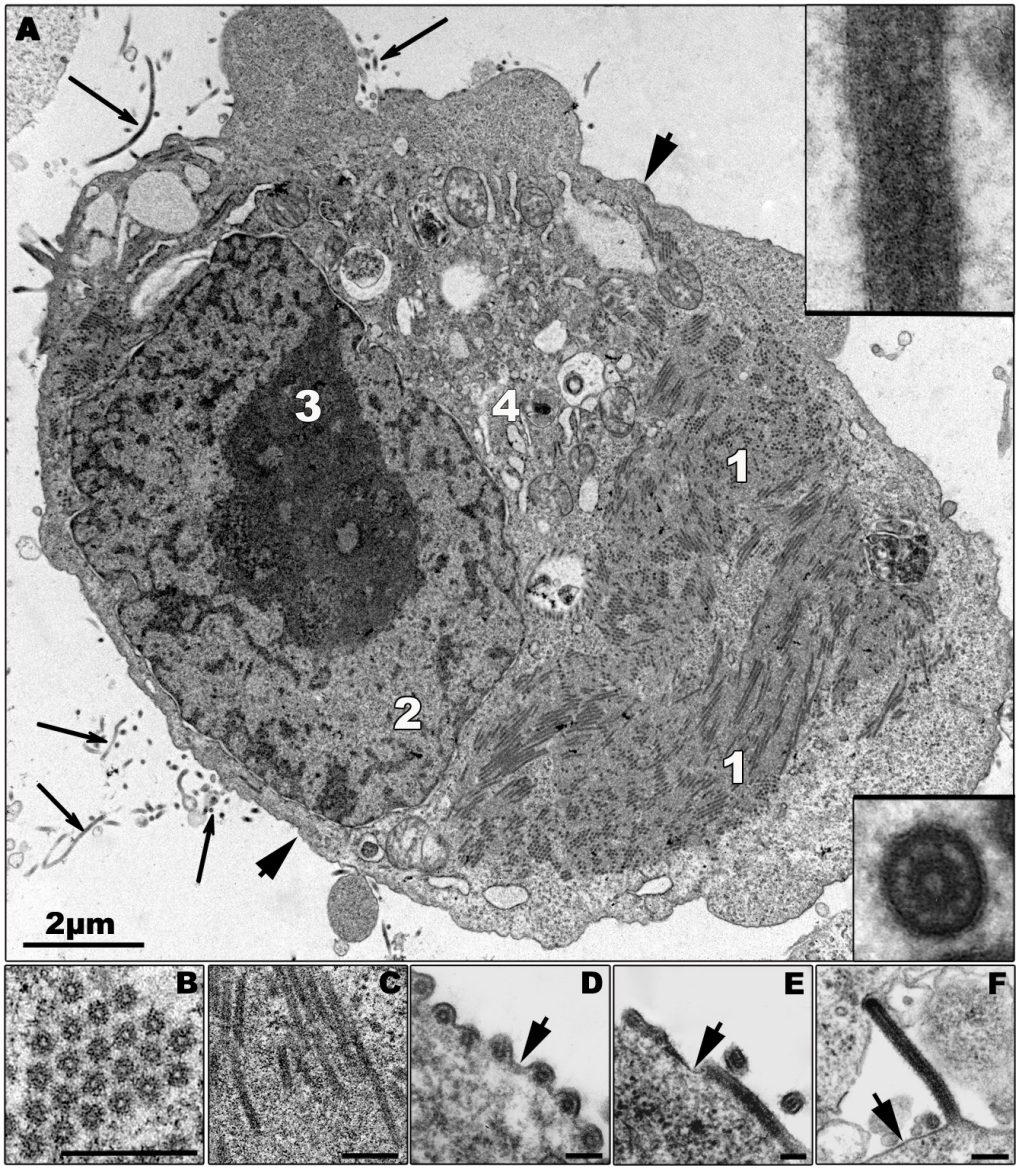
Morphological characteristics of *Zaire ebolavirus* (ZEBOV) replication. (**A**) Ultrathin section of a ZEBOV-infected Vero cell containing large viral inclusions. The inclusions are composed of granular material (1; also shown in (C)) and rod-like nucleocapsids. Released virions are indicated by long arrows and are shown in the inserts. (**B**) Cross section of viral inclusion containing nucleocapsids. (**C**) Longitudinal section of viral inclusion containing nucleocapsids. (**D–F**) Budding of viral particles. In the initial step of budding a particle can be positioned parallel (D and E, cross and longitudinal sections), or perpendicular (**F**) to the membrane and then subsequently is enveloped. Short arrows indicate the cellular plasma membrane. 2—nucleus, 3—nucleolus, 4—Golgi zone. Bars in Figures 2B–F correspond to 250 nm. Thick part of frame around cross-sectioned virion corresponds to 120 nm, and thick part of frame around longitudinal section corresponds to 160 nm. Transmission electron microscopy. Cells were fixed at 16 h p.i.

It is believed that the amplification of the viral genome and the assembly of newly synthesized nucleocapsids occur in highly organized regions in the cytoplasm, the viral inclusions [103,106,107] (Figure 2A). The appearance of granular material of average electron density in the cytoplasm of the infected cells at 12 h (MARV) and 9 h (ZEBOV) post infection (p.i.), respectively, is the first morphological sign of viral replication, as revealed by electron microscopic studies following the course of MARV and ZEBOV infection in Vero and MDCK cells [50]. The granular material is closely associated with the surface of the ER and contains viral proteins and RNA (Figure 2C). Tubular structures with an average diameter of 50 nm then appear in the granular material representing the newly synthesized viral nucleocapsids [50,105] (Figures 2B,C). The viral inclusions can be easily detected as large irregularly formed cytoplasmic aggregates by immunofluorescence microscopy [108,109]. To date, it is not known if cellular components are required for inclusion formation.

**Figure 3.**
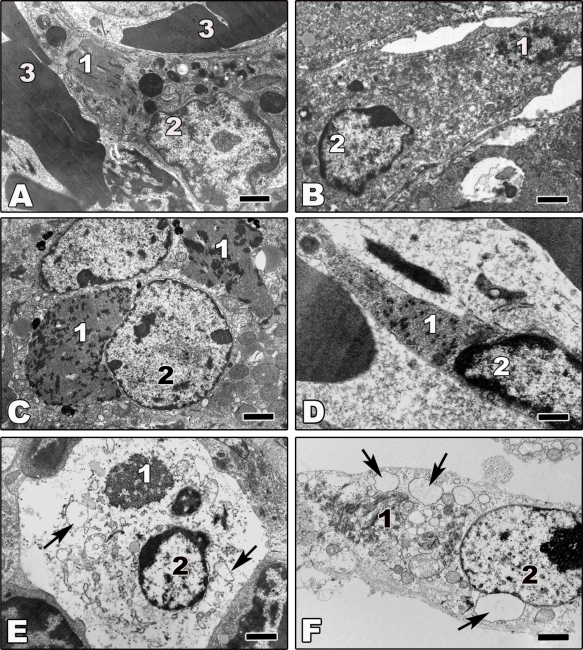
Transmission electron microscopy of ultrathin sections of tissues from animals experimentally infected with filoviruses. Tissues were fixed at day 5 or 6 p.i. (**A**) Ultrathin section of liver tissue from ZEBOV-infected rhesus monkey showing an infected macrophage. (**B**,**C**) Ultrathin sections of liver tissue from Marburg virus (MARV)-infected guinea pig. Shown are an infected macrophage (B) and an infected hepatocyte (C). (**D**) Ultrathin section of spleen tissue from ZEBOV-infected African green monkey showing an infected endothelial cell. (**E**) Ultrathin section of lymphatic node tissue from ZEBOV-infected African green monkey showing a necrotic infected macrophage. (**F**) Ultrathin section of a necrotic ZEBOV-infected Vero cell showing an infected macrophage. Arrows show vacuolization of endoplasmic reticulum cisterns in cells undergoing non-apoptotic cell death. 1—filovirus inclusions; 2—nucleus; 3—erythrocyte. Bars correspond to 2 μm.

The morphological characteristics of filovirus replication in animal cells *in vivo* are identical to those observed in cell culture. We analyzed the morphology of inclusion bodies in EBOV and MARV-infected cells of various organs of non-human primates, guinea pigs, mice, and chick embryos, as well as Vero, BHK-13 and MDCK cells and observed that filovirus inclusions, though morphologically heterogeneous, are always composed of granular material and varying numbers of nucleocapsids [50]. A few examples of filovirus infected cells in animal tissues are presented in Figure 3.

### 3.3. Exit: Rats Abandon a Sinking Ship

Following assembly, newly synthesized nucleocapsids are transported to the sites of virus budding. Immunoelectron microscopy of EBOV-infected cells revealed that NP and the matrix protein VP40 accumulate in the viral inclusions and are closely associated during viral morphogenesis [110]. Meanwhile, it has been confirmed by multiple studies that the filoviral VP40 protein is the major player in viral budding, though various viral proteins significantly enhance the release of viral particles (reviewed in [111–113]). Importantly, filoviruses exploit the vesicular transport machineries of the infected cell for viral egress, and viral proteins of both MARV and EBOV have been reported to interact with various protein components of the COPII vesicular transport system and the ESCRT machinery (reviewed in [111,114,115]). Viral budding occurs either at intracellular membranes, the multivesicular bodies (MVB) [116], or at the plasma membrane [50,105,117]. In cell culture, MARV particles are preferentially released at filopodia, which may facilitate the infection of neighboring cells [117].

Electron microscopic studies of filovirus-infected cells suggested two models for the release of the long, filamentous viral particles: horizontal budding after lateral association of the nucleocapsids with the plasma membrane or vertical budding [50,105,117] (Figures 2D–F). A recent electron tomography study of MARV-infected cells convincingly reconciled the results into a single model. Welsch and colleagues [118] showed that the budding process is initiated by the lateral association of the intracellular nucleocapsids with the plasma membrane. Starting from one end, the nucleocapsids are then subsequently wrapped by the plasma membrane until the viral particles protrude vertically from the cell surface. The formation of such long filamentous particles (700–900 nm or more) is certainly challenging for the infected cells and may lead to membrane perturbation in the cells and in the released viruses. Notably, the release of infectious filamentous MARV from cultured cells has been reported to peak at early time points post infection (1–2 days p.i.), when the cells were still intact. At late time points (4 d p.i.), most of the infected cells were vesiculated, the released virions were morphologically different, being round or bent, and coincidentally infectivity was decreased [118].

## 4. To Live or Die: The Fate of Infected and Non-Infected Cells in Filovirus Infection

### 4.1. Mechanisms of Cell Death

**Figure 4:**
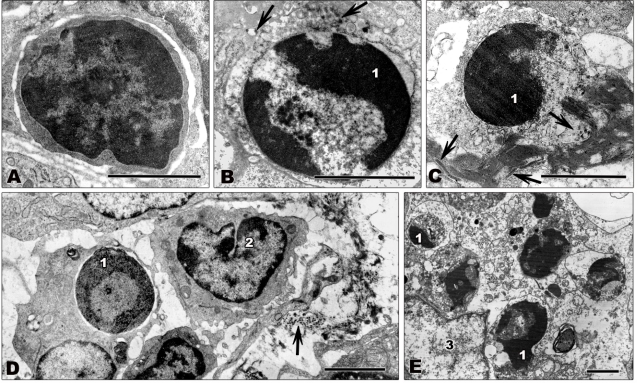
Transmission electron microscopy of ultrathin sections of lymph node tissue from filovirus-infected African green monkeys infected with ZEBOV (**A**,**C–E**) or MARV (**B**). Tissues were fixed at 4 days p.i. (**A**) Small lymphocyte showing normal nuclear morphology with large areas of heterochromatin. (**B**,**C**) Apoptotic lymphocytes. Typical signs of apoptosis such as chromatin condensation and marginal location of chromatin are visible. (**D**,**E**) Apoptotic lymphocytes being engulfed by macrophages. (D) shows initial stages of phagocytosis. The lymphocyte is engulfed by a macrophage. The macrophage shown in (E) contains several destroyed apoptotic lymphocytes. Arrows show filoviral particles. 1—highly condensed heterochromatin; 2—monocyte showing normal nuclear morphology; 3—nucleus of macrophage. Bars correspond to 2 μm.

Viruses exploit the host cell for successful replication, potentially leading to death of the infected cell. Since the production of progeny viruses is hampered when the infected cells are destroyed early in infection, many viruses have evolved mechanisms to avoid host cell death, as reflected by a complex interaction between viruses and cell death signaling pathways (reviewed in [119,120]). Different types of cell death, including apoptosis, necrosis, and autophagy, can be described by different morphological and biochemical characteristics (reviewed in [121,122]). Apoptosis or programmed cell death is characterized by shrinking of the dying cell, plasma membrane blebbing, nuclear condensation, and final fragmentation of the cell in apoptotic bodies [123,124]. Tissue lymphocytes from filovirus-infected non-human primates showing typical signs of apoptosis, such as condensation and marginal localization of chromatin, are shown in Figures 4B–E. Biochemically, apoptosis is characterized by activation of caspases. Caspase cleavage can be activated by extrinsic pathways via death receptor signaling induced by TRAIL, Fas/CD95 (Fas), or TNFα or by intrinsic pathways via regulation of cytochrome C efflux from the mitochondria (reviewed in [125–127]). Lymphocyte apoptosis plays an important role in T cell development and control of T cell tolerance [128] and might also play a crucial role in the pathogenesis of filovirus infection as discussed in Section 4.3. In contrast to apoptosis, necrosis is generally described as uncontrolled cell death, but recent findings suggest that it also might be regulated by conserved biochemical mechanisms (reviewed in [129,130]). Necrosis is characterized by swelling of the cell and cellular organelles, membrane blebbing, vacuolization and results in rupture of the plasma membrane [121,124]. Autophagy is a conserved pathway of eukaryotic cells for recycling cellular components. However, extensive cellular stress can lead to autophagic or type II cell death. Autophagic cells feature vacuolization and formation of double membraned vesicles, the autophagosomes, for degradation of cellular content [131,132]. Characteristic features of non-apoptotic cell death, such as vacuolization, swelling, and the lack of chromatin condensation can be seen in Figures 3E,F.

### 4.2. Infected Cells

In cell culture, filovirus infection leads to a clear cytopathic effect (CPE) including cell blebbing, cell rounding, vacuolization, and detachment [109,118,133,134]. However, the severity of the observed CPE is dependent on the virus species and the cell line used [54,135,136]. The mechanisms leading to cell death in filovirus infection are far from being understood and the published data are to some extent contradictory. While some studies describe apoptotic cell death for isolated primary human macrophages infected with ZEBOV or Bundibugyo [136,137], others did not observe any signs of apoptosis in filovirus-infected primary human cells including monocytes/macrophages from peripheral blood mononuclear cell cultures, macrovascular endothelial cells, or microvascular endothelial cells [54,138].

Electron microscopic studies and biochemical analyses of tissues from infected animals indicate that infected cells, including macrophages, DCs, hepatocytes, and endothelial cells, do not undergo apoptosis [47–50,55,60,62,63,69,138]. Morphologically, the cells appear normal (Figure 3A–D) or they show signs of necrosis (Figures 3E and F). A few examples of filovirus-infected cells from tissues from infected animals are shown in Figure 3. Although the shown tissue macrophages (Figures 3A,B,E,F), hepatocytes (Figure 3C), and endothelial cells (Figure 3D) are infected, as indicated by the presence of viral inclusions in the cytoplasm of the infected cells, they do not show any signs of apoptosis. Vacuolization of the infected cells, however, indicates that they finally undergo non-apoptotic cell death (Figures 3E,F). Notably, the number of swollen and necrotic cells increases during infection [49,55,62,63,69,138].

Intriguingly, the number of DCs was shown to dramatically decrease early in ZEBOV infection in baboons, African green monkeys, and rhesus macaques, and they were totally absent at 6–7 days p.i. [50]. In contrast, DCs were still present in ZEBOV-infected cynomolgus monkeys on day 6 [55]. These differences might be attributed to species-specific variability in the various non-human primate models for filovirus infection [139].

In non-human primates and guinea pigs, infected hepatocytes develop necrosis not related to the formation of inflammatory foci. The liver is a major target organ for filovirus infection and necrotic hepatocytes appear 3–4 days p.i. in numbers that depend on the infectious dose [44,47,50,60,69]. Notably, significant apoptosis of hepatocytes has been observed in a lethal mouse model for ZEBOV and seems to play an important role in pathogenesis; however, it is not clear if the apoptotic hepatocytes were infected [3].

Extensive filovirus infection may lead to the depletion of cellular stocks and disruption of cellular homeostasis. Morphologically, such depleted cells first show signs of swelling and vacuolization of cellular organelles including the ER, mitochondria and Golgi, suggesting that the cells are unable to maintain a normal water-ion balance. This is followed by cellular swelling, vacuolization and cell rounding, which leads to the formation of vacuolar structures consistent with necrosis in the cytoplasm of infected cells (Figures 3E,F), reflecting edematous conditions within the cells [140]. Whether this is due to damage to ion pumps in the plasma membrane or vacuolization of the membrane resulting in cell permeability as a result of viral budding is unclear. As noted earlier, however, the changes in the host cells are also coincident with changes in the progeny virus morphology and infectivity.

#### 4.2.1. Interaction of Filoviruses with Cellular Pathways

The striking lack of infected apoptotic cells reported in most studies raises the question if filoviruses manipulate signaling pathways involved in apoptosis or cell survival. A first hint pointing in this direction has been given by a recent study showing that EBOV entry leads to the activation of the PI3K/Akt signaling pathway very early in infection, resulting in the activation of Rac1, a regulator of endocytosis and vesicular trafficking [141]. PI3K is embedded in a complex network of signaling cascades regulating cell metabolism, proliferation and survival, and many viruses modulate the PI3K/Akt pro-survival pathway to prevent premature apoptosis of infected cells [142]. The temporal modulation of PI3K activation during viral infection is critical, and different arms of the PI3K signaling network might be activated by distinct viral triggers during the replication cycle. Thus, it has been shown for influenza virus that transient PI3K activation during entry leads to the activation of Rac1, while late in infection, PI3K activation prevents the induction of premature apoptosis [143]. It is currently not known if PI3K/Akt pro-survival signaling cascades are activated at late stages in filovirus infection, but this would be an attractive model to at least partially explain why apoptosis does not seem to be the preferred mechanism of cell death in filovirus infection.

Filoviruses are known to interfere with antiviral signaling pathways. Since the innate immune response to filovirus infection and the corresponding viral countermeasures are the main subject of another review article in this issue, we will focus on the mechanisms involved in the regulation of apoptosis. To combat viral invasion, cells have evolved multiple antiviral defense mechanisms that are activated upon infection and ideally, will lead to the elimination of the viral intruders. The antiviral response is initiated by a cellular detection process mediated by various cellular pattern recognition receptors (PPR) that specifically recognize pathogen-associated molecular patterns (PAMPs). Typical PAMPs of negative-sense RNA viruses are surface proteins and RNA [144,145]. A prominent filovirus PAMP are the 5′triphosphate ends of the genomic RNA which can be sensed by RIG-I, leading to the induction of the type I interferon (IFN) response [146]. However, in cells infected with EBOV, the induction of the type I IFN response via RIG-I activation is blocked by the polymerase cofactor VP35 (reviewed in [147,148]). Recent findings show that RIG-I activation may lead to the induction of mitochondrial-mediated apoptosis, which is triggered by binding of the IFN regulatory factor 3 (IRF-3) to the pro-apoptotic Bax protein. The IRF-3-mediated induction of apoptosis has been considered an important antiviral defense mechanism against RNA viruses [149–151]. In addition, it has been reported that the mitochondrial antiviral signaling protein (MAVS; also known as IPS-1, VISA, or Cardif), an adaptor protein in the RIG-I signaling pathway, is able to activate apoptosis signaling, and this activation can be inhibited by viruses to prevent the induction of apoptosis [152,153]. Although one can speculate that the inhibition of RIG-I signaling by VP35 may prevent the induction of apoptosis in infected cells, no data are available to support this hypothesis. Noteworthy, the activation of PPRs by viral infection or double-stranded (ds) RNA may likewise induce anti-apoptotic signaling through NFκB, resulting in increased host cell survival [154–156] (reviewed in [157]).

The antiviral protein dsRNA-dependent protein kinase R (PKR) senses dsRNA and is a main regulator of IFN signaling and apoptosis. Since dsRNA is formed during replication and transcription of multiple RNA and DNA viruses [158], it is considered to be a major PAMP in viral infection. Upon activation by dsRNA PKR phosphorylates the translation initiation factor eIF2α, leading to translational arrest and apoptosis [159]. Intriguingly, PKR is not activated in ZEBOV-infected cells, and VP35 has been shown to actively block PKR activation [160,161]. It has been reported for various viruses that they inhibit PKR-mediated eIF2α phosphorylation to prevent host cell apoptosis [162–164]. However, it is not known if the inhibition of PKR in EBOV-infected cells blocks the induction of apoptosis.

Toll-like receptors (TLRs) are another important group of PRRs recognizing viral PAMPs [145]. TRL signaling was found to play pro- and anti-apoptotic roles in different cell types highlighting its role in regulation of host cell death [165,166]. The EBOV surface protein GP is recognized by TLR4 and activates TLR4 signaling, including activation of NFκB [167]. It remains unclear, however, if TLR4 signaling is triggered in the context of EBOV infection, since in a recent study, EBOV VP35 was found to interfere with the maturation of DCs induced by lipopolysaccharide, an agonist of TLR4 [168]. VP35 was also shown to inhibit the induction of IFN in murine DCs after treatment with CpG DNA, an agonist for TLR9 [169]. However, a recent study by Leung *et al.* [170] suggests that cells utilizing TLR-mediated antiviral pathways such as plasmacytoid dendritic cells are less prone to the inhibitory effects of VP35 EBOV than cells relying on RIG-like signaling pathways. In conclusion, the role of TLR signaling in filovirus infection is not well understood and additional studies using filovirus infection models are needed.

#### 4.2.2. The Cytopathic Factor GP

Multiple expression studies have implicated the EBOV surface protein GP in cytotoxicity and cell damage, inducing cell rounding, detachment, and membrane permeabilization [92,171–177], while other EBOV proteins did not induce cell detachment [171]. Expression of GP also leads to a general downregulation of cell surface proteins including adhesion molecules, MHC class I proteins, and EGF receptor [92,174,176,178]. Moreover, GP expression in explanted blood vessels resulted in endothelial cell loss and increased vascular permeability [171].

As mentioned above, GP is proteolytically cleaved in two subunits, GP_1_ and GP2. Intriguingly, both GP subunits contribute to the observed cytopathogenicity. The cytopathic domain within GP_1_ has been mapped to the so-called mucin-like domain, a highly O- and N-glycosylated serine-threonine-rich region of about 150 amino acids in length, which is sufficient to cause detachment and downregulation of cellular surface proteins [171,178,179]. Interestingly, the mucin-like domain does not only induce cytopathic effects in cells expressing EBOV GP, but was also shown to induce activation of NFκB and ERK signaling pathways in cells treated with EBOV-like particles [180].

It has been suggested that the GP-induced cytotoxic effects are caused by the interaction of GP with the GTPase dynamin, leading to interference with the intracellular trafficking of cell surface proteins [176]. However, a recent study has questioned the involvement of dynamin in GP-induced CPE [178]. GP has also been shown to modulate the ERK/MAPK signaling cascade by reducing ERK2 activation. This effect is dependent on the mucin-like domain. Since downregulation of active ERK2 leads to a decreased alphaV-integrin expression, which is associated with cell rounding and detachment, it has been suggested that ERK2 signaling cascades are involved in the induction of GP-mediated cytopathic effects [179]. Another possible mechanism of GP-induced cytotoxicity might be the induction of ER stress associated with the unfolded protein response. A recent study reports that ectopically expressed GP containing the mucin-like domain accumulates in the ER, whereas GP lacking the mucin-like domain is distributed throughout the cell and does not localize in the ER. When the membrane-anchored GP_2_ subunit is expressed in cells in the absence of GP_1_, it also induces cytopathic effects by enhancing the permeability of the plasma membrane. This effect is mediated by the transmembrane domain [177,181]. If and how the different proposed mechanisms to explain GP-mediated cytopathogenicity are connected, is currently not known.

There is some debate over the ability of GP to induce cell death. Several groups reported that expression of GP, though inducing detachment of the cells, does not lead to cell disruption [172,174], while others observed cell death [171,175,179]. It is possible that the observed differences are due to experimental differences, including GP expression rates, the expression system used, long-term *versus* short-term expression, and the cell type. Intriguingly, GP has been shown to induce a specific form of apoptosis, anoikis, in primary human cardiac microvascular endothelial cells, while THP-1 cells, a human monocyte–macrophage-derived cell line, did not undergo cell death upon GP expression [175]. Another group has shown that GP-induced cell death is non-apoptotic [179].

Since the described data are based on ectopic expression of GP in the absence of other viral proteins, the question arises: What happens in the infected cells? Volchkov *et al.* [173] addressed this question by using a recombinant EBOV system. The membrane-anchored full-length GP is synthesized from an edited version of the GP mRNA, which accounts for about 20% of the total GP mRNA. When the editing site was mutated in the viral genome, all GP mRNA molecules were translated into the full-length version of GP, leading to the production of enhanced levels of membrane-anchored GP. The mutant was significantly more cytopathogenic than wild-type virus, indicating that the expression rate of GP in infected cells determines its cytotoxic properties. These results were confirmed by more recent studies showing that low expression rates of GP helped to avoid cytotoxic effects [133]. In addition, there might be other regulatory mechanisms in infected cells to mitigate GP-induced cytotoxicity. As mentioned above, VP35 is able to block various signaling pathways involved in the antiviral response to viral infection and it is conceivable that VP35 also interferes with cellular signaling pathways involved in GP-induced cellular stress.

It remains puzzling that MARV GP does not seem to be cytotoxic when expressed in the absence of other viral proteins, although it is as highly glycosylated as EBOV GP [172]. Also, infection studies in non-human primates revealed that the endothelium remains relatively intact even late in infection, suggesting that the hemorrhagic symptoms in EBOV infection are not caused by EBOV-induced cytolysis of infected endothelial cells, but are rather the result of dysregulated immune mechanisms targeting the vascular system [54,182,183]. Endothelial cells are secondary target cells in filovirus infection and the morphological characteristics of filovirus replication in these cells do not differ from those in other tissues or in cell culture [47,50,51,54,110].

### 4.3. Non-Infected Cells

Lymphocytes do not support filoviral replication, perhaps because they lack receptors for these viruses [184,185]. Nevertheless, lymphocyte apoptosis is a characteristic feature of filovirus infections and can be observed in both blood and tissues of infected patients and animals [7,55,138,186–188]. Figure 4 shows apoptotic lymphocytes in lymph node tissue from filovirus-infected animals. The apoptotic lymphocytes are not infected and are engulfed by surrounding macrophages. Filovirus-induced lymphocyte apoptosis is also observed in EBOV-infected human PBMCs [137]. The apoptosis of non-infected lymphocytes is not unique to filoviruses. It has been reported in a number of viral infections, including lymphocyte choriomeningitis virus [189], human immunodeficiency virus [190], human herpesvirus 6 [191] and Vaccinia virus infections [192]. Although protection from apoptosis of infected cells is a strategy for survival for a number of viruses [193], induced periods of transient or chronic lymphocytopenia brought about by bystander apoptosis is thought to contribute to the generalized immunosuppression that accompanies some viral infections [192,194].

As mentioned above, apoptosis can be initiated by both extrinsic factors and intrinsic mechanisms, both of which activate caspases and lead to DNA fragmentation and cell death (reviewed in [195]). The best known example of bystander lymphocyte apoptosis in viral infections is in CD4 T cells during HIV infection. Nevertheless, despite years of study, the mechanisms of depletion of these cells remain controversial. However, extrinsic and intrinsic pathways appear to be involved. Although CD4 T cells are a target of HIV, the majority of lymphocytes that are depleted are uninfected, and the depletion of uninfected cells accelerates with disease progression [190,196]. Both viral proteins released from infected cells [197–199] and host-derived proteins [200,201] have been suggested to contribute to CD4 T cell death. More recently, an accumulation of incomplete viral transcripts during abortive infection of resting CD4 T cells has been demonstrated to activate intrinsic pathways that lead to apoptosis during HIV infection [202]. It is unclear which of these mechanisms account for the majority of T cell apoptosis during HIV infection, or if multiple mechanisms are important in HIV pathogenesis. Given that many gaps remain in our understanding of bystander T cell apoptosis in HIV infection, despite years of study, it is not surprising that we would have even less of a universal view of how filoviruses cause bystander cell loss.

In the case of filovirus infection, not only are CD4 T cells depleted, but CD8 T cells and NK cells as well [7,55,186], suggesting that some generalized mechanisms may contribute to lymphocyte apoptosis in filovirus disease. Using a mouse model of EBOV infection, Bradfute *et al.* [3] suggested that both intrinsic and extrinsic apoptotic pathways may contribute to lymphocyte depletion. If this is also the case for the disease in humans and non-human primates, then it is likely that multiple mechanisms contribute to the generalized lymphocyte depletion. The loss of lymphocytes has been postulated to contribute to the failure to generate fully protective adaptive immune responses in these species [7,203–205]. In fact, extensive lymphocyte apoptosis has been shown to proceed the generation of adaptive responses in humans with fatal disease [7], making this hypothesis all the more plausible.

EBOV-infected cells may secrete TRAIL and increased levels of soluble Fas have been detected in the sera of some EBOV-infected non-human primates [55,206]. Moreover, increased expression levels of TRAIL and Fas mRNA have been observed in peripheral blood mononuclear cells of infected non-human primates [55]. These could trigger conventional extrinsic pathways of apoptosis in susceptible cells, including T cells. This view is supported by an analysis of Fas expression on T cells from patients who survived or succumbed to EBOV infection [188]. In patients who died, a very high proportion of the few remaining CD4 and CD8 T cells were Fas positive in contrast to T cells from survivors. Although Fas can be induced as a result of specific T cell activation [207], it is unlikely that the residual cells in these patients were only antigen-specific T cells.

It is also possible that dysregulated DCs and macrophages could contribute in other ways to lymphocyte apoptosis. Infected DCs and macrophages fail to produce regulated cytokine responses, and they are also impaired in their ability to upregulate costimulatory molecules, such as CD40 and the B7 family member CD86, consistent with their inability to efficiently prime T cells [208,209]. Interestingly, however, other members of the B7 family, including co-inhibitory molecules such as programmed death ligand 1 (PD-L1) are upregulated on infected DCs, and its receptor, programmed death 1 (PD-1) may be upregulated on CD8 T cells in EBOV infections [210]. The interaction of these molecules could lead to apoptosis. The PD-1/PD-L1 pathway is important for controlling T cell tolerance and has been shown to be used by pathogens to down-regulate T cell responses [128,211]. PD-1 signaling results in decreased T cell proliferation and recent findings suggest this might be due to induction of apoptosis via PD-L1 binding [212,213]. While this might explain the depletion of antigen-specific T cell populations, this mechanism is unlikely to explain the generalized lymphopenia observed during filovirus infection, since the infected DCs would not be expected to engage T cells that were not specific for filovirus epitopes sufficiently to trigger PD-1 signaling.

Alternatively, T cell apoptosis may result in part from the dysregulated cytokine responses during filovirus infections. IFNγ helps productively modulate CD8 T cell responses if present at or near the time of T cell activation. However, if not, IFNγ can have pro-apoptotic consequences on T cells [214]. Interestingly, IFNγ is present at high levels in the serum late in fatal filovirus infection [2,162], and these high levels could result in the activation of T cell and/or NK cell apoptotic pathways. Finally, a 17-mer in filovirus GPs, which resembles an immunosuppressive motive found in retroviral envelope proteins, has been reported to induce lymphocyte death and suppression of cytokine responses [215–217]. This is puzzling, because lymphocytes do not bind to the viral GP [184,185]. So the mechanism by which the 17-mer mobilizes lymphocyte death remains unclear.

## 5. Conclusions

We have learned a lot about the interaction of filoviruses with infected (and non-infected) cells, including identification of target cells, elucidating stages of the replication cycle, cytopathic effects induced by the viruses, and host restriction factors. Nevertheless, there is still a long way to go towards understanding both the cellular and organismal events that lead to the outcome of this devastating disease. There remain major gaps in our understanding of the alterations in signaling pathways in infected cells, the cellular components that contribute to filovirus replication, and the mechanisms that control cell fate in infected cells. Moreover, we remain largely ignorant in our understanding of the mechanisms that impact the innate and adaptive immune system and modulate inflammatory responses during infection. These avenues of inquiry will be crucial for us to have a more complete understanding of the pathogenesis of filoviral disease.
